# The Influence of COVID-19 Lockdown Restrictions on Perceived Nutrition Habits in Rugby Union Players

**DOI:** 10.3389/fnut.2020.589737

**Published:** 2020-10-26

**Authors:** Charlie Roberts, Nicholas Gill, Stacy Sims

**Affiliations:** ^1^Te Huataki Waiora School of Health, University of Waikato, Hamilton, New Zealand; ^2^New Zealand Rugby Union, Wellington, New Zealand

**Keywords:** COVID-19, Rugby Union, athlete, nutrition habits, training, social distancing measures

## Abstract

The global outbreak of COVID-19 has led to governments and local authorities implementing nationwide lockdowns in an attempt to encourage social distancing and minimize the spread of the virus. Only essential businesses have been able to remain open, with non-essential businesses and activities either closing or restricting services. With no group training sessions allowed, canceled matches, an inability to work and the closure of eating establishments, Rugby Union players have experienced disruption to their daily lives. Two surveys were distributed among Rugby Union athletes to explore (1) the influence of COVID-19 lockdown restrictions on Rugby Union players' nutrition and training habits and (2) how nutrition habits in New Zealand Rugby Union players change after lockdown restrictions were lifted. In total, 258 respondents completed Survey 1 (84.1% male, 26.4% professional/semi-professional). Of the respondents, 58% indicated they lived with family during lockdown. Total food intake was reported to be higher in 36% of respondents. Fruit and vegetable intake was lower (17%) and packaged/convenience food intake higher (26%) in a minority of respondents. In total, 106 respondents completed Survey 2 (84.9% male, 34.0% professional/semi-professional). Of the respondents, 72% prepared and 67% purchased their own food. Less than half of respondents consumed high-protein food more than twice daily either during or following lockdown. Compared to during lockdown, motivation to train and exercise was greater in 58% of respondents following lockdown. Dieticians and nutritionists within clubs provided most of the nutrition knowledge to athletes however other unreliable sources were identified, such as social media and family members. The ongoing pandemic has presented significant challenges for athletes concerning training and nutrition habits and the current study provides some insight into these. Coaches and performance staff should ensure athletes receive appropriate nutritional and training support whilst being aware of the unique demands the individuals' may face.

## Introduction

COVID-19 is a novel strain of coronavirus that can cause severe acute respiratory distress. It is spread via droplets generated by sneezing, coughing or talking and is therefore easily transmitted between humans. COVID-19 was declared a global pandemic by the World Health Organization on 11th March 2020 (1) and measures have been implemented by governments and local authorities to minimize the spread of the virus. These measures include social distancing (aiming to keep people from different households separate unless required) and rigid hygiene protocols (wearing masks when out in public, regular washing and sanitizing of hands and surfaces). The implementation of these guidelines has since resulted in global travel bans and restrictions on engaging in activities deemed “non-essential.”

In New Zealand, Alert Level Four lockdown restrictions were enforced between 25th March 2020 and 27th April 2020, with businesses that aren't deemed essential (e.g., supermarkets, pharmacies) forced to close. Between 27th April 2020 and 13th May 2020, Alert Level Three was enforced in New Zealand which brought around some changes (e.g., fast food establishments could operate a take-away service only) however gatherings of more than 10 people from different households was not permitted. Following the reduction to Alert Level Two on 13th May 2020, which allowed for most restaurants, businesses and activities to resume provided appropriate contact tracing, social distancing and hygiene rules were enforced, most people were able to resume habitual activities. On 8th June 2020, Alert Level One meant no restrictions were enforced in New Zealand with the exception of border entry being strictly controlled to allow only citizens and permanent residents entry following a 2 week quarantine.

Organizations and individuals involved in sport and physical activity have experienced significant restrictions due the COVID-19 social distancing measures. Sporting events at all levels, from schools and clubs to large international events such as the 2020 Olympic Games, have been canceled or postponed. This has resulted in organized training sessions being deemed non-essential with many athletes forced to reduce their training volume drastically. Additionally, stresses related to illness and health, economic uncertainty and prolonged social isolation may lead to unfavorable or additional mental health outcomes in athletes (2, 3).

Engaging in regular physical activity has been encouraged (4) although social distancing guidelines may affect team and contact sports. Although some athletes are able to continue training and preparing for competition, sports requiring physical contact face additional issues surrounding training preparation. Rugby Union is a combative sport and the high incidence of players engaging in physical contact in the form of tackles, rucks, mauls, and scrums (5) thus social distancing guidelines have particular relevance to collision-based team sport athletes. Rugby Union athletes have been unable to participate in sport-specific activity during lockdown restrictions, which vary from country to country. Strict lockdown measures restricting access to habitual training environments and contact with other players mean coaches and practitioners are faced with challenges regarding developing or maintaining essential attributes required by Rugby Union athletes (lean mass, strength, power, speed, agility, sport-specific skills, decision-making ability) (6).

Not only have training and competition been affected by COVID-19, lockdown restrictions are likely to have influenced athletes' perceived nutrition habits. Nutrition habits can be defined as “the habitual decisions of individuals or group of people regarding what foods they eat” (7). For example, habitual eating patterns will have changed significantly with the closure of eating establishments and food delivery services. Furthermore, stressors as a direct result of COVID-19 lockdown restrictions may result in unfavorable food choices (8). Additional challenges associated with monitoring nutrient intake during lockdown are likely to be encountered by clubs due to less direct contact with nutrition professionals. It is vital athletes receive the appropriate nutritional support during lockdown as an abrupt return to training and play once restrictions are lifted may result in increased risk of injury (9) and it is likely good dietary habits may alleviate this (10, 11).

Numerous surveys have been distributed across different populations and countries to identify the effect of COVID-19 lockdown measures on dietary patterns (12, 13) however no data has been reported in Rugby Union athletes. The purpose of this study is to explore (1) the influence of COVID-19 lockdown restrictions on Rugby Union players' nutrition and training habits and (2) how nutrition habits in New Zealand Rugby Union players change after lockdown restrictions were lifted.

## Methods

The research instruments consisted of two surveys administered via an online survey-hosting website (Survey Monkey, Palo Alto, CA, USA). Within human ethics research regulations, this study was deemed to be low risk. All participants granted informed consent prior to commencing any of the surveys.

The first survey, entitled “Nutrition & Training Habits in Rugby Union Players – COVID-19 Lockdown” was administered from April 19th 2020 to May 22nd 2020, during which full lockdown restrictions were enforced in New Zealand and many countries globally. Initially, management staff and/or coaching staff from Rugby Union teams located in New Zealand, Australia and the United Kingdom were approached via e-mail. The purpose of the survey was explained, and management staff were asked to distribute the survey web link to their players. The survey consisted of 30 questions organized into three sections—general information, nutrition and training.

The second survey, entitled “Post-Lockdown Nutrition & Training Habits in Rugby Union Players” was administered from 2nd June 2020 to 2nd July 2020. The survey consisted of 28 questions. Management staff from Rugby Union teams in New Zealand were approached via e-mail due to the complete relaxation of lockdown restrictions. An information page was presented prior to the survey and consent was gained via a consent statement and check box.

Descriptive analysis of results are presented as percentages (%) of responses.

## Results

For a full breakdown of Survey 1 and 2 questions and responses, please see Supplementary Materials. Relevant results will be discussed in the subsequent sections.

### Survey 1

Relevant results will be discussed in this section.

In total, of the 314 survey link clicks, 258 respondents (82% total) completed Survey 1. Demographics for the survey respondents are presented in Table 1. The respondents were 18–25 years of age (59.7%), male (84.1%) and living in New Zealand (92.2%). Only 26.3% of respondents reported playing at either a semi-professional or a professional level, with amateur players representing competitive club level or academy teams. Living with family accounted for 58.5% of responses. During lockdown, 39.2% of respondents indicated their motivation to train was lower than usual, with 32.6% reporting no change in motivation levels and 28.3% stating motivation to train had increased during lockdown.

**Table 1:** Demographic information for survey 1 (during lockdown) and survey 2 (after lockdown).

		**During Lockdown**	**After Lockdown**
**Question**	**Groups**	***N* (%)**	***N* (%)**
Age	<18 years	14.3	17.9
	18–25 years	59.7	66.0
	26–35 years	21.3	16.0
	>35 years	4.7	0.00
Sex	Male	84.1	84.9
	Female	15.1	15.1
	Prefer not to say	0.8	0.00
Country of Residence	New Zealand	92.2	100.0
	Australia	3.5	0.0
	United Kingdom	4.3	0.0
How would you describe your living situation before lockdown restrictions were enforced?	Alone	2.3	–
	With a partner	13.2	–
	With family (parents, siblings, etc.)	36.8	–
	With family (partner, children, etc.)	9.7	–
	With friends	4.7	–
	Flatting	26.4	–
	Other	7.0	–
How would you describe your current living situation?	Alone	1.9	2.8
	With a partner	13.2	17.9
	With family (parents, siblings, etc.)	58.5	33.0
	With family (partner, children, etc.)	9.7	2.8
	With friends	3.9	7.6
	Flatting	10.1	31.1
	Other	2.7	4.7
How would you best describe your level of play?	Professional	9.0	17.0
	Semi-professional	17.3	17.0
	Amateur	73.7	66.0

Most respondents reported consuming breakfast daily (63.2%), eating 2–3 meals (67.1%) and 1–2 snacks (63.6%) per day. Respondents reported that a family member was most likely to purchase their food during lockdown (42.6%). Whilst 44.6% of respondents prepared their own food and meals, family members (23.6%) and a combination of people (23.3%) (the respondent and others in the household) were also involved in the food/meal preparation process.

Total food intake was reported to be greater during lockdown in 35.7% of respondents. During lockdown, 16.7% of respondents' fruit and vegetable intake was lower than before lockdown, with the remainder indicating their intake was either the same or higher than before the restrictions. Packaged/convenience food intake was lower in lockdown for 41.9% of participants.

Nutrition knowledge in respondents was primarily from a dietician or nutritionist associated with the club (61.6%) however coaching staff (25.2%), teammates (27.1%), family members (30.6%), the internet (31.1%) and social media also contributed. When asked about the frequency of consumption of complete high-protein foods, 49.2% of respondents reported greater than two daily feedings. Respondents who consumed no dietary supplements during lockdown consisted of 53.3% of total responses.

### Survey 2

In total, of the 112 survey link clicks, 106 respondents (95% total) completed Survey 2. Demographics for the survey respondents are displayed in Table 1. Respondents were 18–25 years old (66.0%) and male (84.9%). In total, 34.0% of respondents perform at a semi-professional or professional level. Most respondents reported living with family (33.0%) or flatting in shared accommodation (31.1%). Motivation to train and exercise was greater in 59.5% of respondents compared to during lockdown.

As with Survey 1, most respondents reported daily breakfast consumption (72.6%), eating 2–3 meals (51.9%) and 1–2 snacks (71.7%) per day. The vast majority of respondents purchased (67.0%) and prepared their own food and meals (71.0%). Eating out at takeaways, cafes and restaurants at least once per week was reported in 45.3% of respondents, with 8.4% indicating their frequency of eating out was ≥3 times weekly.

Dieticians or nutritionists associated with the club provided 68.9% of respondents nutrition knowledge, with coaching staff (26.4%), teammates (33.0%), family members, the internet (31.1%) and social media (22.6%) again contributing. Consumption of ≤1 complete, high-protein food source daily was reported by 50.9% of respondents. No supplement use was reported in 32.1% of respondents.

### Comparison Between Survey 1 and Survey 2

To compare results between surveys, responses from athletes located in countries other than New Zealand were filtered out. Relevant results are displayed in Figures 1–8 as comparisons between results presented by amateur players and semi-professional/professional players.

**Figure 1:**
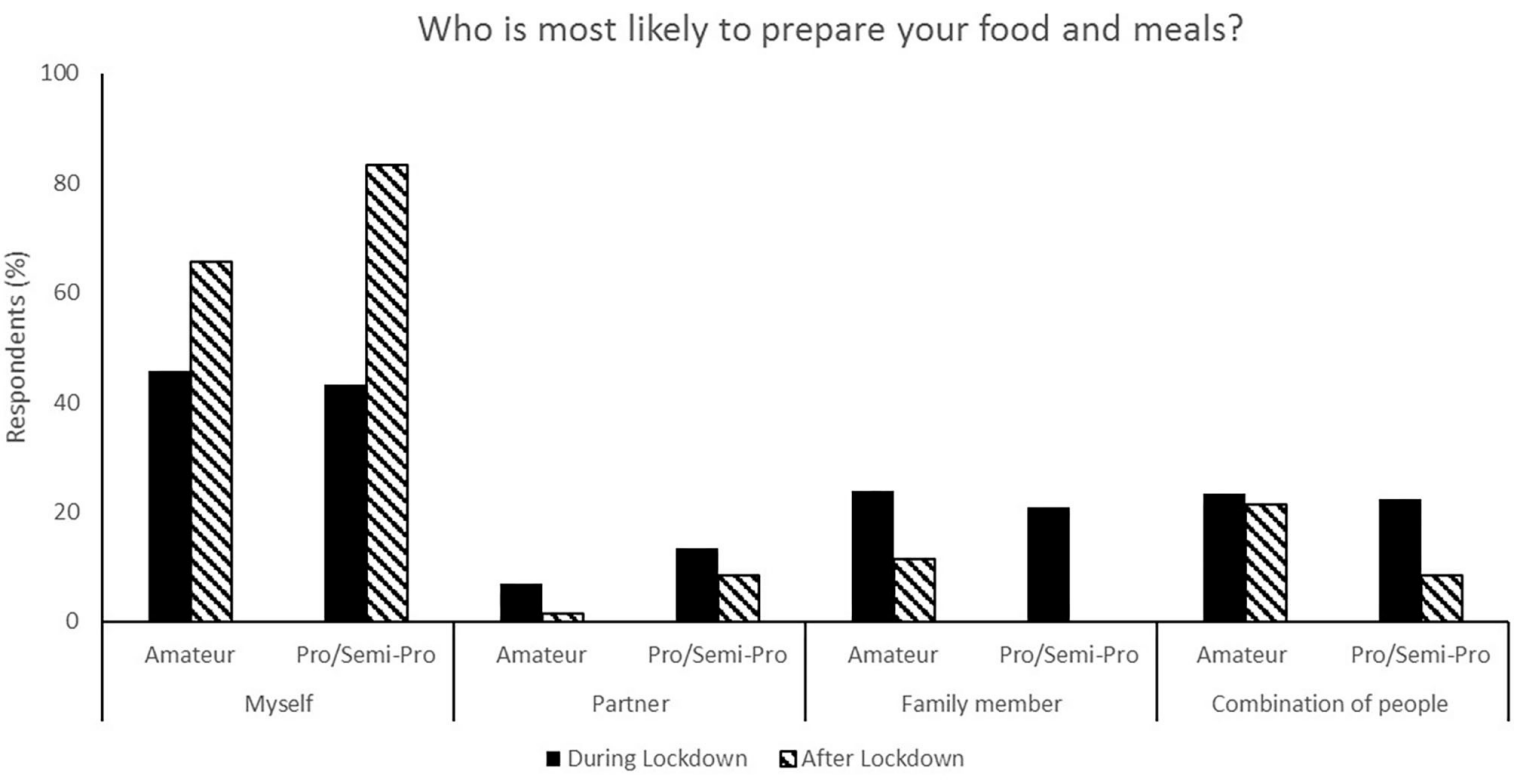
Responses for food and meal preparation during (survey 1) and after (survey 2) lockdown.

**Figure 2:**
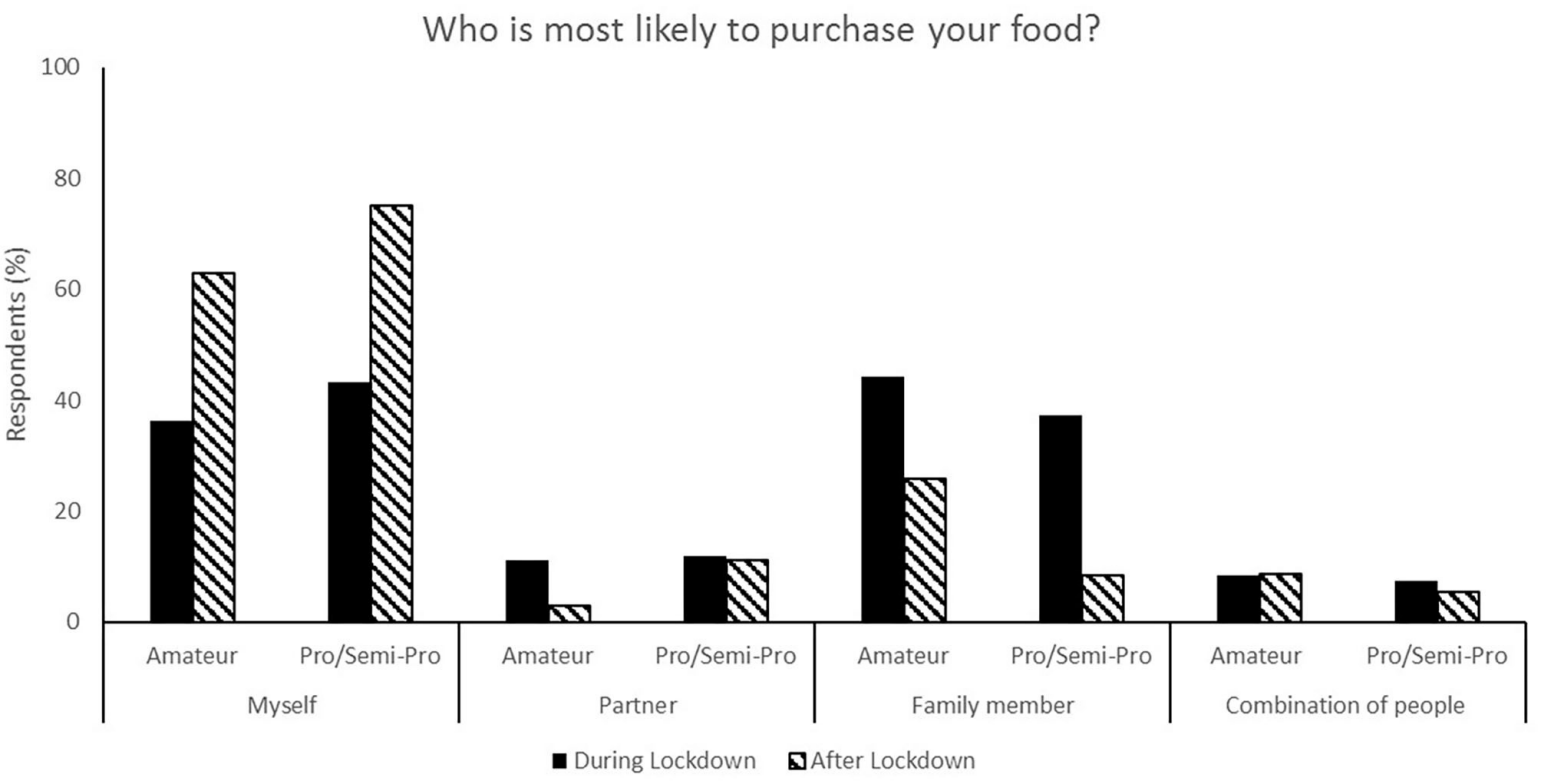
Responses for food purchasing during (survey 1) and after (survey 2) lockdown.

**Figure 3:**
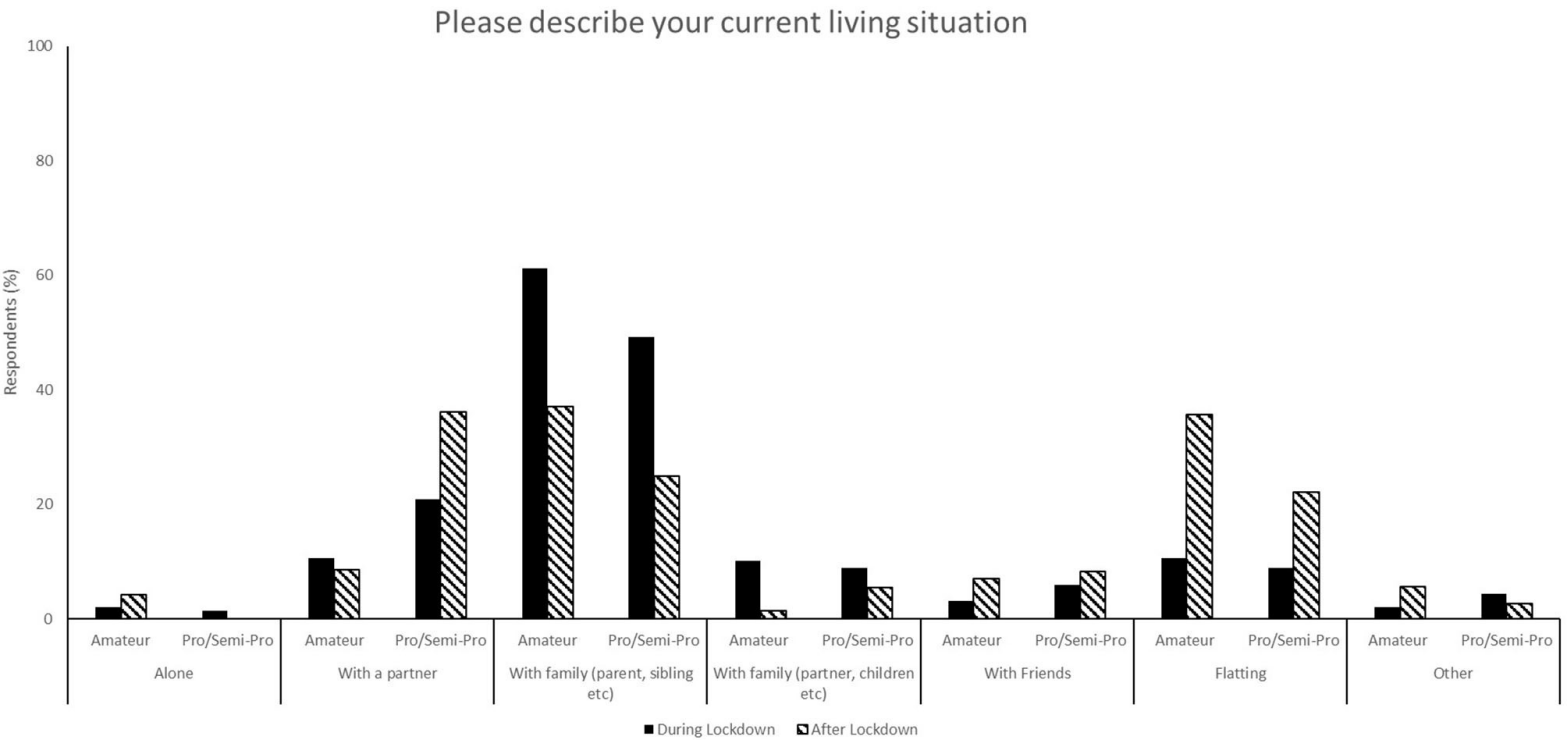
Responses for living situations during (survey 1) and after (survey 2) lockdown.

**Figure 4:**
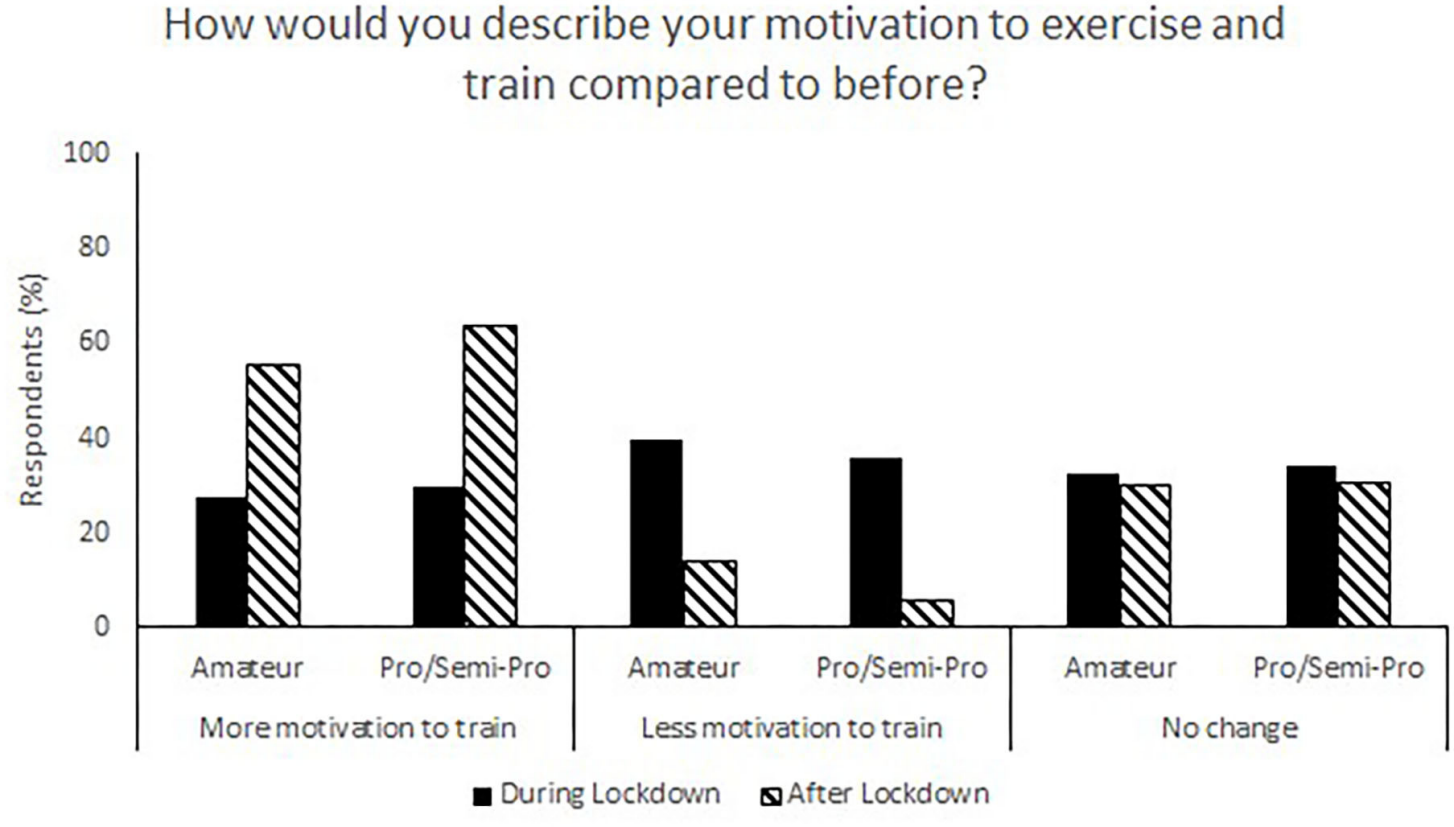
Responses for motivation to train during (survey 1) and after (survey 2) lockdown.

**Figure 5:**
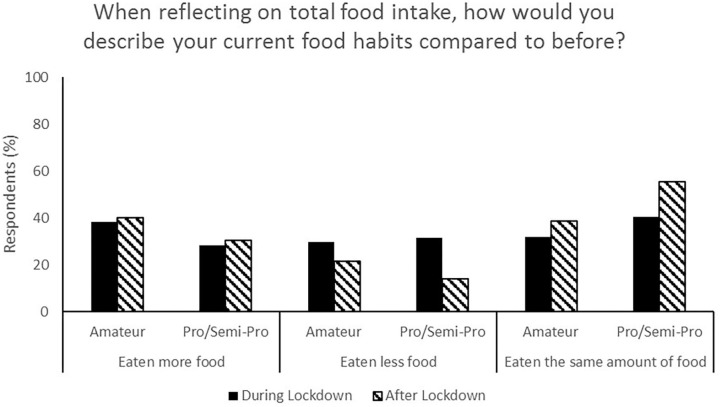
Responses for total food intake during (survey 1) and after (survey 2) lockdown.

**Figure 6:**
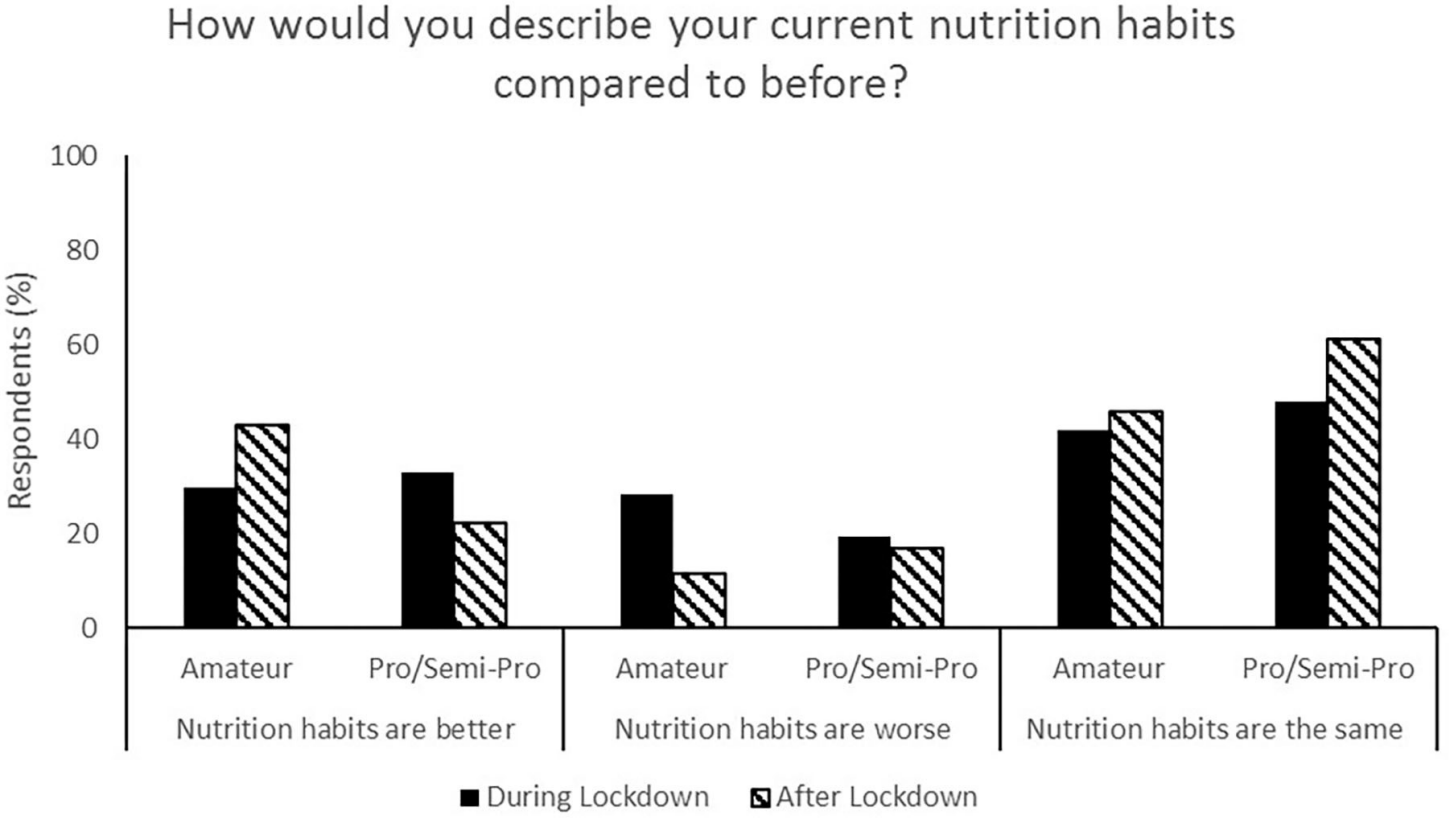
Responses for nutrition habits during (survey 1) and after (survey 2) lockdown.

**Figure 7:**
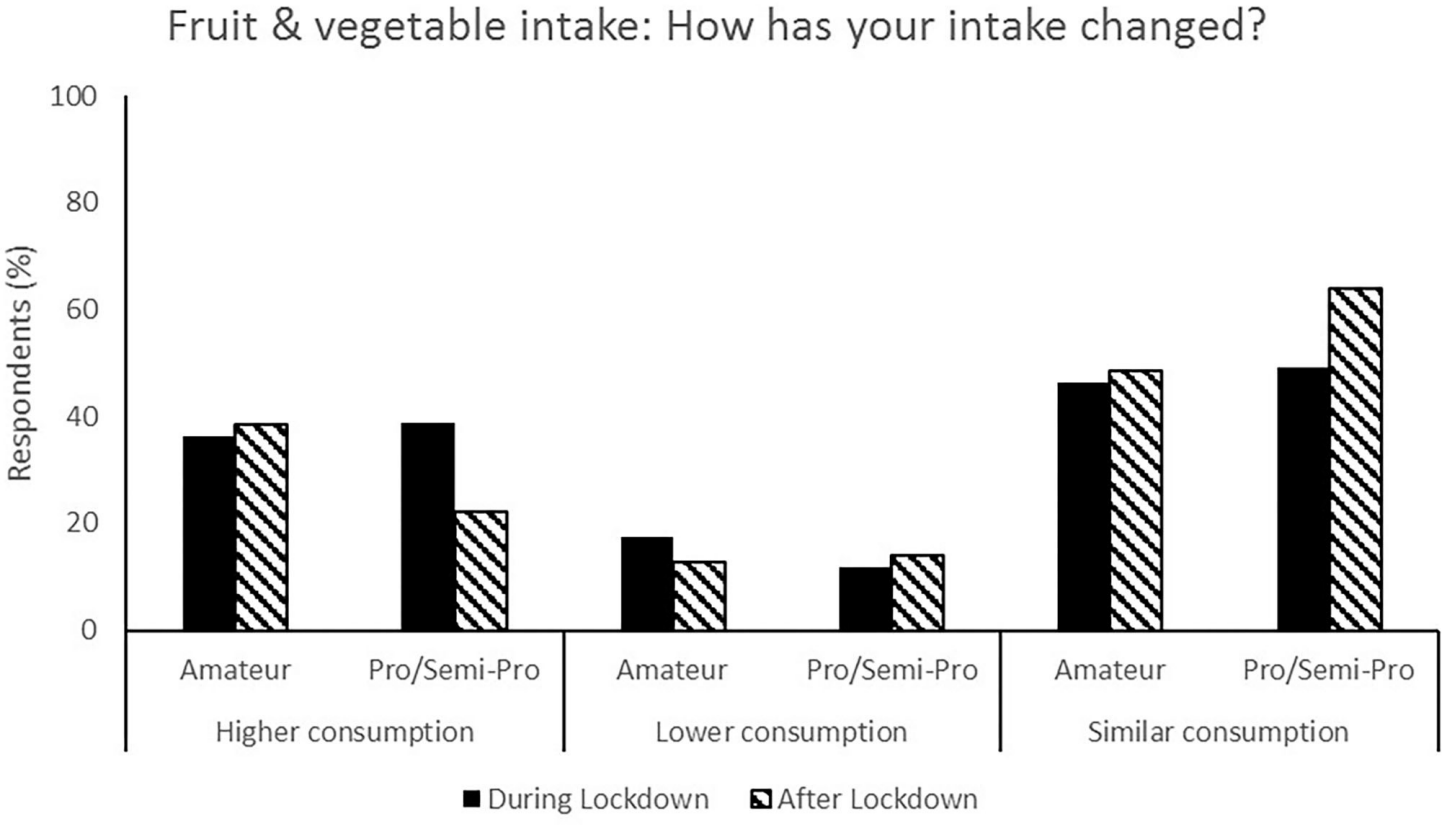
Responses for fruit & vegetable intake during (survey 1) and after (survey 2) lockdown.

**Figure 8:**
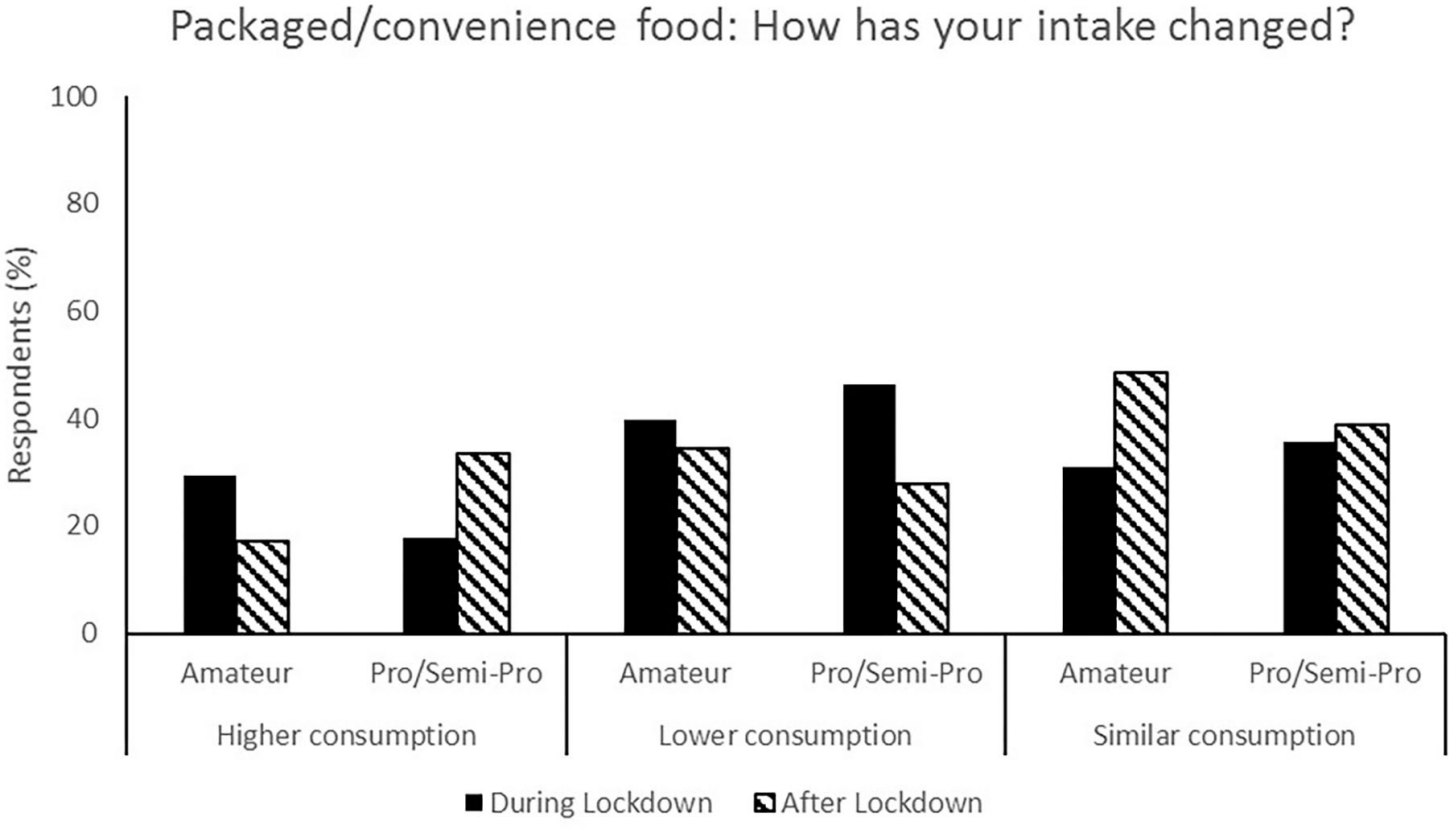
Responses for packaged/convenience food during (survey 1) and after (survey 2) lockdown.

The relaxation of lockdown restrictions caused a change in living situations. Living with family accounted for 58.5% of responses during lockdown compared to 33.0% following lockdown, and flatting in shared accommodation accounted for 10.1% of responses during lockdown and 31.13% after lockdown (Figure 3). Following lockdown, the responses indicate a shift to athletes becoming more self-reliant and both purchasing (67.0% compared to 38.0%, Figure 2) and preparing (71.7% compared to 44.6%, Figure 1) their own food and meals. Post-lockdown, respondents reported a much higher motivation to train (57.9%) compared to during lockdown (28.7%) (Figure 4).

Most respondents reported no change in nutrition habits from during lockdown to post-lockdown (50.9%) whilst 35.9% indicated that their nutrition habits were better (Figure 6). Additionally, total food (44.3%, Figure 5), fruit and vegetable (53.8%, Figure 7) and packaged/convenience food (45.3%, Figure 8) was reported to be the same following lockdown.

## Discussion

The purpose of this study was to explore (1) the influence of COVID-19 lockdown restrictions on Rugby Union players' nutrition and training habits and (2) how nutrition habits in New Zealand Rugby Union players were affected by the relaxation of lockdown restrictions. Nationwide lockdowns are unprecedented and as such no data is currently available describing how the combinations of (1) disruption to daily life (2) inability to train and eat habitually and (3) stress due to the global pandemic may have influenced these factors in athletes.

### During Lockdown

The majority of responses indicated that their food intake during lockdown either remained the same or increased. Whilst the long-term implication of COVID-19 lockdown restrictions on body composition have not been reported, athletes would be wise to reduce total energy intake to reflect the reduction of physical activity (14). Many respondents engaged in regular training sessions (89.4% reported completing ≥3 sessions per week) however the shift from habitual practices likely resulted in a large reduction in daily energy expenditure. Factors influencing energy expenditure during lockdown in athletes include a requirement to perform training sessions either at home or in local outdoor areas such as parks, a lack of equipment (free weights, machines) and no competition between teammates being present. These factors mean minimal rugby-specific training sessions were performed.

The temporary closure of restaurants, fast food outlets, cafes and bars may have resulted in better food choices in athletes. Most respondents indicated that fruit and vegetable intake during lockdown either remained the same or increased from pre-lockdown. Additionally, packaged and convenience food intake either remained the same or was lower during lockdown. These contrast with general population surveys distributed during COVID-19 lockdown restrictions, with one third of 1097 Polish respondents not consuming fresh fruit or vegetables on a daily basis (13). In a global survey distributed through 35 research institutions, respondents reported eating in an unhealthier pattern along with consuming more snacks and meals when COVID-19 lockdown restrictions were enforced (15).

Ingesting adequate protein (1.2–2.0 g.kg.d) (16) is an important factor in ensuring lean mass retention. When inadequate dietary protein is consumed, negative protein balance can result in muscle protein catabolism, adversely affecting muscle mass and function (17). In Survey 1, only 43.9% of respondents reported consuming a whole-food, high biological value protein source more than twice daily, which may not be optimal for retaining lean mass during a prolonged lockdown period.

During lockdown, most of respondents implied they consumed no dietary supplements. High heterogeneity exists in the evidence-base surrounding dietary supplement use among athletes and prevalence recorded between studies is variable (18). Additionally, consultation with a nutrition professional should occur to consider the benefits and risks involved with consuming certain supplements (19). Nonetheless, consumption of certain supplements may be beneficial for athletes provided training, nutrition and recovery habits are sound (20). Due to the additional stressors of the lockdown, supplement use may not have been a priority for athletes.

Protein supplementation is a beneficial strategy for athletes to reach daily requirements (21). Whilst protein intake is recommended to primarily come from whole foods, whey and casein are of a high quality, and consumption is considered safe and convenient (16). With most survey respondents reporting consuming a high-protein food source ≤ once daily, this may be a useful strategy for athletes to minimize the detrimental effects of a reduction in training volume, a lack of appropriate equipment and limited rugby-specific training (6).

### Post-lockdown

As many athletes appeared to return to the family home for the lockdown period, there would likely be a greater reliance and/or sharing of responsibilities surrounding nutrition. Following lockdown, those staying with family appeared to decrease and those flatting in shared accommodation increased. Therefore, there was a large shift in respondents indicating becoming more self-reliant, with a large number of athletes reporting purchasing their own food and preparing meals themselves. Reliance on others to prepare and cook meals can be a major barrier to healthy eating in athletes when those preparing meals do not possess appropriate sports nutrition knowledge. Furthermore, athletes cooking/shopping skills and cost can present further challenges in athletes at all levels of play (22). These factors should be considered by sports dieticians/nutritionists when aiming to improve athletes' nutrition knowledge and food intake.

Sports nutrition knowledge in respondents from both surveys appeared to mainly come from dieticians or nutritionists associated with the clubs however a large number also reported other sources including other individuals (coaching staff, teammates, family members) or through seeking it themselves (internet, social media). Many coaches provide nutrition advice to athletes however these individuals do not often possess the appropriate level of knowledge required to do so (23). Teammates and family members who have not received appropriate training can provide incorrect and potentially harmful information. Additionally, it is important that athletes are aware of the possibility of unreliable information being presented through digital channels (24).

Fast food outlets, cafes and takeaways were able to re-open following the relaxation of lockdown restrictions in New Zealand. A number of athletes reported eating out on >3 occasions weekly. Eating out can indeed be incorporated into an optimal diet for an athlete however it is important for these individuals to be aware of how to make appropriate choices. Increased frequency of fast-food consumption is associated with poorer diet quality, perhaps due to displacement of appropriate food choices (25). Less frequent food preparation and more frequent fast food consumption are associated with poorer diet quality, however time-restraints are a major influence on these factors (26). As most respondents identified as amateurs, it is likely these athletes also had full-time obligations in the form of work and/or studies. Furthermore, the social aspect of being involved in a team may outweigh focusing on eating for optimal performance, recovery and health in amateur athletes (27).

Unsurprisingly, most athletes indicated their motivation to exercise and train was higher once lockdown restrictions were relaxed. The physical and aggressive nature of the game, on and off-field interactions with teammates and feelings of achievement and success are some of the factors previously reported to increase participation motivation in elite female Rugby Union athletes (28). Lockdown restrictions would indeed cause major disruption to all of these factors. Additionally, the closure of habitual training facilities (clubhouses, gyms) and stressors associated with the pandemic are likely to have influenced athletes' motivation to train during lockdown.

Respondents of both Surveys 1 and 2 reported consuming high biological-value protein sources from whole foods less than once per day. These responses may indicate a lack of knowledge of the importance of regular protein consumption throughout the day for athletes. As with during lockdown, athletes concerned with maximizing lean mass and strength gains are recommended to consume a minimum of 1.6 g.kg.d spread evenly across at least 4 meals of 0.4 g.kg (29–31). Although athletes during lockdown will be experiencing different levels of exercise stimulation (lack of resistance training equipment, minimal rugby-specific training) which likely resulted in reductions in lean mass, strength and skill adequate protein consumption may allow for a faster return to pre-lockdown body composition and performance levels. Minimizing lean mass catabolism is also an important factor in reducing injury risk when Rugby Union training and match play resumes, with the development of the muscle tissue important for withstanding external forces associated with collision sport play (6).

### Limitations and Conclusion

A major limitation of the present study is that the accuracy of the responses cannot be verified. Due to the anonymous nature of the online surveys distributed for this study, responses may not truly indicate how a respondent feels. Additionally, the nature of the questions means accurate results are difficult to obtain (for example, asking participants whether their food intake has changed as a result of lockdown measures) and information on macronutrient intake other than protein was not requested. Furthermore, respondents may not answer truthfully due to the additional stressors resulting from COVID-19, such as economic struggles, feelings of isolation or health worries.

Participants were not asked to describe the nature of training sessions performed during lockdown, which presents another major limitation. It is likely that the implementation of lockdown measures would adversely affect resistance training and sport-specific training in athletes. With no access to commercial gym equipment, athletes are required to make use of limited equipment they may have at home or potentially none at all. If inadequate resistance training is performed, changes associated with muscle disuse such as lean mass and strength loss and increased fat mass may rapidly occur (32). It has been suggested that low-volume and low-intensity contractions are adequate at stimulating muscle protein synthesis and preventing muscle wasting (33). Resistance training protocols utilizing bands and bodyweight exercises have previously demonstrated efficacy in promoting lean mass gains in healthy older individuals (34) however this is not likely to be enough to maintain strength in Rugby Union athletes. Indeed, athletes are unlikely to have access to the equipment required to perform key exercises for developing or maintaining maximal strength (multi-joint resistance exercises such as squats and deadlifts) (35) at sufficiently heavy loads (≥75% 1-repetition maximum) (36). For optimal performance and injury prevention, Rugby Union athletes are expected to possess high levels of strength and power whilst engaging in high metabolic training volumes and rugby-specific sessions (37) and re-building these attributes before competitive matches resume will be crucial. Most respondents indicated they completed at least three training sessions per week however no information was gathered as to the nature of these sessions and as such, the ability of the respondents to offset muscle disuse wasting cannot be predicted.

In conclusion, the COVID-19 pandemic and associated restrictions to encourage social distancing and delay the spread of the virus have presented significant challenges for athletes of all levels and disciplines. Appropriate nutritional and training support may assist athletes retain adequate performance, lean mass, strength and cardiorespiratory fitness during lockdown scenarios however no data is currently available to support this. Coaches and performance staff can assist athletes by promoting greater protein intakes and feeding frequency, encouraging safe supplement use and keeping players engaged in interesting training sessions that can be completed from home. Staff must also be aware of the challenges athletes may be facing, related or unrelated to the ongoing pandemic.

## Data Availability Statement

The raw data supporting the conclusions of this article will be made available by the authors, without undue reservation.

## Ethics Statement

Ethical review and approval was not required for the study on human participants in accordance with the local legislation and institutional requirements. The patients/participants provided their written informed consent to participate in this study.

## Author Contributions

CR, NG, and SS contributed to the development and distribution of the survey materials and edited the manuscript. CR drafted the manuscript. All authors contributed to the article and approved the submitted version.

## Conflict of Interest

The authors declare that the research was conducted in the absence of any commercial or financial relationships that could be construed as a potential conflict of interest.
