# Supplementary material for: The Influence of COVID-19 Lockdown Restrictions on Perceived Nutrition Habits in Rugby Union Players
Source: Front Nutr. 2020 Oct 26;7:589737. doi: 10.3389/fnut.2020.589737 (PMC7649138; doi:10.3389/fnut.2020.589737)
Supplement: Supplementary file 1 [file Table_1.DOCX]

|  |  | During Lockdown | After Lockdown |
| --- | --- | --- | --- |
| Question | Groups | N (%) | N (%) |
| **How often do you consume breakfast?**  **How many meals do you eat per day?**  **How often do you eat snacks?**  **Who is most likely to prepare your food and meals?**  **Who is most likely to purchase your food?**  **My knowledge about nutrition comes from (choose all that apply)**  **How would you rate your nutrition habits now compared to before lockdown? (SURVEY 1)**  **How would you rate your nutrition habits now compared to during lockdown? (SURVEY 2)** | Every day  4-6 days per week  1-3 days per week  Never   - 1. per day   2-3 per day  ≥ 4 per day  ≤ 1 per day  1-2 per day  3-4 per day  ≥ 5 per day  Myself  Partner  Family Member  Combination of people  Myself  Partner  Family Member  Combination of people  Dietician/nutritionist associated with the club  Dietician/nutritionist not associated with the club  Coaching staff  Teammates  Friends not with team  Family member  Internet  Social Media  Television  No nutrition knowledge  Other  Nutrition habits have been better  Nutrition habits have been worse  Nutrition habits have been the same | 63.2  16.3  15.1  5.4  1.2  67.1  31.8  4.7  63.6  29.1  2.7  44.6  8.5  23.6  23.3  38.0  11.2  42.6  8.1  61.6  17.4  25.2  27.1  16.7  30.6  34.5  24.4  5.8  4.3  18.2  30.6  26.0  43.4 | 72.6  18.9  5.7  2.8  0.0  51.9  46.1  2.8  71.7  25.5  0.0  71.7  3.8  7.6  17.0  67.0  5.7  19.8  7.6  68.9  21.7  26.4  33.0  16.0  32.1  31.1  22.6  2.8  1.9  9.4  35.9  13.2  50.9 |
| **When reflecting on total food intake, how would you describe your current food habits compared to before lockdown? (SURVEY 1)**  **When reflecting on total food intake, how would you describe your current food habits compared to during lockdown? (SURVEY 2)** | Eaten more food  Eaten less food  Eaten the same amount of food | 35.7  30.2  34.1 | 36.8  18.9  44.3 |
| **Fruit & vegetable intake: How has your intake changed since lockdown restrictions were implemented? (SURVEY 1)**  **Fruit & vegetable intake: How has your intake changed since the relaxation of lockdown restrictions? (SURVEY 2)**  **Packaged/convenience food: How has your intake changed with since lockdown restrictions were implemented? These types of foods may include ready meals, chocolate bars, cakes, crisps, processed meat products and fries(SURVEY 1)**  **Packaged/convenience food: How has your intake changed with relaxation of the lockdown restrictions? (SURVEY 2)**  **How often did you consume alcohol DURING lockdown? (SURVEY 1 & 2)**  **How often did you consume alcohol BEFORE lockdown? (SURVEY 1)**  **How often have you consumed alcohol AFTER lockdown? (SURVEY 2)**  **Which of the following foods have you eaten during lockdown? (SURVEY 1)**  **Which of the following foods have you eaten since lockdown restrictions were relaxed? (SURVEY 2)**  **How often have you eaten ANY of the above during lockdown? (SURVEY 1)**  **How often have you eaten ANY of the above since lockdown restrictions were relaxed? (SURVEY 2)**  **How often are you consuming dietary supplements?**  **How would you describe your motivation to exercise and train during lockdown compared to before? (SURVEY 1)**  **How would you describe your motivation to exercise and train since lockdown restrictions were relaxed compared to during lockdown? (SURVEY 2)**  **How many training sessions did you have BEFORE lockdown? (SURVEY 1)**  **How many training sessions did you have DURING lockdown? (SURVEY 2)**  **How many training sessions have you had DURING lockdown? (SURVEY 1)**  **How many training sessions have you had since lockdown restrictions were relaxed? (SURVEY 2)** | Higher consumption during lockdown  Lower consumption during lockdown  Similar consumption during lockdown  Higher consumption during lockdown  Lower consumption during lockdown  Similar consumption during lockdown  Every day  A few times per week  About once per week  A few times per month  ≤ once per month  Never  Every day  A few times per week  About once per week  A few times per month  ≤ once per month  Never  White meat  Red meat  White fish  Oily fish  Eggs  Low-fat dairy products  Legumes  Tofu  ˃ 3 times per day  2-3 times per day  Once per day  Most days per week  A few days per week  ≤ Once per week  Never  ˃ Once per day  Once per day  Most days per week  A few times per week  Rarely  Never  More motivated to train  Less motivated to train  No change in motivation levels  None  1-2 per week  3-4 per week  5-6 per week  ˃ 6 per week  None  1-2 per week  3-4 per week  5-6 per week  ˃ 6 per week | 36.4  16.7  46.9  26.4  41.9  31.8  1.2  12.0  18.2  20.5  19.0  29.1  0.4  5.4  26.7  29.5  21.7  16.3  95.7  93.8  65.5  34.1  91.9  91.5  39.2  7.8  11.6  37.6  18.2  26.7  4.7  0.8  0.4  10.1  14.7  5.8  7.4  8.9  53.1  28.3  39.2  32.6  1.6  7.4  20.9  44.6  25.6  5.0  5.4  29.1  35.3  25.2 | 33.0  13.2  53.8  22.6  32.1  45.3  0.9  4.7  17.0  20.8  24.5  32.1  0.0  4.7  18.9  32.1  20.8  23.6  86.8  91.5  53.8  30.2  84.9  78.3  38.7  5.7  8.5  40.6  17.0  24.5  6.6  2.8  0.0  11.3  22.6  14.2  8.5  11.3  32.1  58.5  11.3  30.2  0.0  8.5  24.5  45.3  21.7  0.0  4.7  23.6  45.3  26.4 |
|  |  |  |  |
|  |  |  |  |
|  |  |  |  |
|  |  |  |  |
